# Intraspecific Trait Variation and Coordination: Root and Leaf Economics Spectra in Coffee across Environmental Gradients

**DOI:** 10.3389/fpls.2017.01196

**Published:** 2017-07-12

**Authors:** Marney E. Isaac, Adam R. Martin, Elias de Melo Virginio Filho, Bruno Rapidel, Olivier Roupsard, Karel Van den Meersche

**Affiliations:** ^1^Department of Physical and Environmental Sciences and Centre for Critical Development Studies, University of Toronto Scarborough, Toronto ON, Canada; ^2^Department of Geography, University of Toronto, Toronto ON, Canada; ^3^Centro Agronómico Tropical de Investigación y Enseñanza Turrialba, Costa Rica; ^4^CIRAD, UMR SYSTEM Montpellier, France; ^5^CIRAD, UMR Eco&Sols Montpellier, France

**Keywords:** agroecology, agroforestry, *Coffea arabica*, functional traits, intraspecific trait variation, leaf economics spectrum, root economics spectrum, trait coordination

## Abstract

Hypotheses on the existence of a universal “Root Economics Spectrum” (RES) have received arguably the least attention of all trait spectra, despite the key role root trait variation plays in resource acquisition potential. There is growing interest in quantifying intraspecific trait variation (ITV) in plants, but there are few studies evaluating (i) the existence of an intraspecific RES within a plant species, or (ii) how a RES may be coordinated with other trait spectra within species, such as a leaf economics spectrum (LES). Using *Coffea arabica* (Rubiaceae) as a model species, we measured seven morphological and chemical traits of intact lateral roots, which were paired with information on four key LES traits. Field collections were completed across four nested levels of biological organization. The intraspecific trait coefficient of variation (cv) ranged from 25 to 87% with root diameter and specific root tip density showing the lowest and highest cv, respectively. Between 27 and 68% of root ITV was explained by site identity alone for five of the seven traits measured. A single principal component explained 56.2% of root trait covariation, with plants falling along a RES from resource acquiring to conserving traits. Multiple factor analysis revealed significant orthogonal relationships between root and leaf spectra. RES traits were strongly orthogonal with respect to LES traits, suggesting these traits vary independently from one another in response to environmental cues. This study provides among the first evidence that plants from the same species differentiate from one another along an intraspecific RES. We find that in one of the world’s most widely cultivated crops, an intraspecific RES is orthogonal to an intraspecific LES, indicating that above and belowground responses of plants to managed (or natural) environmental gradients are likely to occur independently from one another.

## Introduction

Elucidating the key dimensions of functional trait variation among plant species has been critical for understanding, predicting, and managing terrestrial ecological responses to environmental or land-use change. Supporting the application of functional trait-based approaches to terrestrial ecology is arguably one unifying framework: evaluating how traits covary or trade-off to form distinct trait “spectra” (Grime, 1979; Lambers and Poorter, 1992; Reich et al., 1997; Westoby et al., 2002; Reich, 2014; Diaz et al., 2016). In addition to early mentions of plant strategies, trait suites, and trait trade-offs (Grime, 1979; Givnish, 1988; Lambers and Poorter, 1992; Chapin et al., 1993), the concept of trait spectra was popularized in the 2000s, most notably with the publication of the “Leaf Economics Spectrum” (LES) (Wright et al., 2004). Shortly before and since the LES was published, nearly all groups of functional traits have been hypothesized to covary along a distinct spectrum that describes functional variation among plant species (Westoby et al., 2002; Chave et al., 2009; Reich, 2014; Diaz et al., 2016). Evidence on trait spectra in plants has been critical for evaluating the evolutionary pressures driving trait variation among species (e.g., Shipley et al., 2006), and understanding how differences in species traits influence ecosystem structure and function (e.g., Diaz et al., 2004; Cornwell et al., 2008).

Among all traits, hypotheses on the existence of a universal “Root Economics Spectrum” (RES) has received arguably the least attention (Reich, 2014), despite the key role root trait variation plays in resource acquisition potential (Lambers et al., 2008; Cahill et al., 2010; Bardgett et al., 2014), and other ecosystem functions such as soil stability (e.g., Rillig et al., 2015). Evidence for the existence of a community-level RES has emerged, with certain traits covarying along a spectrum from resource acquiring to resource conserving root traits. Distinct root morphological and chemical traits [i.e., high specific root length (SRL), specific root area (SRA), specific root tip density (SRTD) (or branching intensity, fine root tip density, and root tip abundance), and root N concentration] designate resource acquisition while large root diameter and high root C:N signify resource conservation (Prieto et al., 2015; Fort et al., 2016; Weemstra et al., 2016). However, studies testing for the presence of a RES across species have produced mixed results. For example, recent studies have found evidence for a RES in herbaceous species, but analyses suggest woody species show drastically different patterns (Larson and Funk, 2016; Roumet et al., 2016; Weemstra et al., 2016).

Even less well resolved, is whether or not there exists a within-species RES, unrelated to genetic diversity but driven by environmental factors. The expression of root plasticity, and thus measurable root intraspecific trait variation (ITV), may be owing to within-species genetic variability or the integration of signals from the rooting environment (Miner et al., 2005). Yet, artificial selection during domestication can limit root trait variation, such that ITV from genetic sources are constrained. Here, we employ two highly related varieties from the economically important tree-crop breeding program for *Coffea arabica*. Given this, our study focuses near solely on phenotypic plasticity derived from the effects of environmental conditions on root trait values and root-leaf trait coordination. Recent analyses of ITV point to within-species trait spectra (Albert et al., 2010; Siefert et al., 2015) as having a key role in determining plant community composition (Laughlin et al., 2012), plant responses to environmental change (Jung et al., 2014; Moran et al., 2016), and rates of ecosystem function (Lecerf and Chauvet, 2008; Gagliardi et al., 2015) (but see Jackson et al., 2013). However, to date this literature has almost exclusively focused on leaf traits. In comparison, to our knowledge there is only one study that directly tests for the presence of a within-species RES. Hajek et al. (2013) found that certain root traits covaried strongly along a single axis of variation that, when coupled with relative growth rate, leaf size, and specific leaf area (SLA), described nearly 70% of the variation in root chemistry and morphology among *Populus trema* individuals. Ostonen et al. (2007) illustrated the relationships between SRL and fertilization within tree species through meta-analysis. Beyond this, there is no evidence indicating whether or not a RES applies to individuals of other species.

Documenting systematic responses of traits to environmental conditions has long been a main theme when evaluating inter- and intraspecific variation in traits (e.g., Givnish, 1988; Chapin et al., 1993). Understanding how plants integrate signals from highly heterogeneous soil environments in order to modify root traits, is arguably more challenging as compared to other aboveground traits. Unlike well-described relationships between light availability and rates of C assimilation and associated traits in leaves (e.g., Rijkers et al., 2000), root traits respond to a range of regional and localized soil chemical resources including macro- and micro-nutrients, soil moisture regimes, and soil pH, as well as multiple physical properties including structure, texture, and aggregation (reviewed by McCormack et al., 2015; Weemstra et al., 2016). These factors would be expected to vary most widely across broad growing regions, as compared to individual-plant scales; an assumption that is widely reflected in many process-based models of agricultural yield and ecosystem services, where trait values are generalized across growing regions (e.g., Bouman and van Laar, 2006; van Oijen et al., 2010). In this sense quantifying the primary sources of variation in traits, especially root traits that are difficult to collect, is key for ensuring that trait values are actually reflective of the plants growing in a particular region, site, or management scenario, and for designing trait sampling strategies that adequately capture the main aspects of ITV (Carmona et al., 2015; Martin et al., 2017).

Another key question in trait-based research is whether or not different trait spectra are “parallel” vs. “orthogonal” (independent of) to one another (Baraloto et al., 2010; Weemstra et al., 2016). Studies on interspecific trait variation have found the LES is orthogonal to suites of whole plant (Diaz et al., 2016), stem (Baraloto et al., 2010), and leaf hydraulic traits (Li et al., 2015). With respect to root traits, studies have found evidence of parallel coordination between RES and other spectra, but these results have been limited to herbaceous species (Craine et al., 2005; Tjoelker et al., 2005), or have been observed only when evaluating trait differences among plant communities (de la Riva et al., 2016).

To date, hypotheses on trait spectra orthogonality within species have been only weakly tested, especially for root traits. One study did find evidence of orthogonality among LES and leaf hydraulic traits in a *Eucalyptus* genotype (Blackman et al., 2016), while the analysis by Hajek et al. (2013) indicates that only certain leaf traits (i.e., leaf area and SLA) covary along a within-species RES, while other leaf traits (i.e., leaf N) do not. Drawing on literature from trees and tree-crops, we expect root ITV to occur along environmental gradients, namely soil moisture (Moser et al., 2010; Padovan et al., 2015) and edaphic conditions (Isaac et al., 2014; Defrenet et al., 2016); gradients that may occur independently of those driving aboveground ITV, such as air temperature, light, and CO_2_ availability (Gagliardi et al., 2015; Niinemets, 2015; Blackman et al., 2016). Additionally, mycorrhizal associations, a key biotic aspect of soils, may lead to systematic differences in ITV of root traits as compared to other trait spectra (Collins et al., 2016).

Testing hypotheses on trait coordination or orthogonality has applied implications for both agroecosystem models and crop biology. From a modeling perspective, if above- and belowground traits coordinate along single axes of resource acquisition/conservation, data on aboveground traits (which are generally easier to collect) could be used to approximate root trait data (that are generally more difficult to ascertain). Alternatively, evidence of orthogonality among root and leaf traits within species would indicate that root trait values are essential when describing, or modeling the impacts of, independent belowground dimensions of plant functional biology such as nutrient capture and retention (Meister et al., 2014). From a crop science perspective, researchers have pointed to the difficulties in artificially selecting crops for desirable root traits (Meister et al., 2014). If root and leaf show coordinated patterns of ITV, managing the environmental conditions that are known to influence leaf traits, would also be expected to influence root traits. Alternatively, if suites of traits vary independently from one another, multiple environmental gradients would have to be managed in order to reach desired functional trait profiles of crops of crop assemblages.

Our study was designed to evaluate patterns of intraspecific root trait variation within an agroecosystem. We use individuals of *C. arabica*, grown in four climatically different sites across two management systems (monoculture and agroforestry) in a nested design to determine primary sources of trait variation. As one of the world’s most widespread tree-crops, ITV of *C. arabica* LES traits have long been a focus on agronomists (e.g., DaMatta, 2004; Matos et al., 2009), and have more recently been evaluated to test hypotheses regarding ITV. For example, Martin et al. (2017) report that while *C. arabica* plants differ along the LES, high-resource agricultural environments lead this species to express weakened patterns of LES trait covariation (when compared to wild plants). Similarly, an analysis by Gagliardi et al. (2015) found that the position of coffee plants along an “intraspecific LES” was associated with plant yield. So while evidence indicates *C. arabica* follows along the LES, and that this variation has implications for agroecosystem functioning, tests on the patterns of *C. arabica* root trait covariation are considerably less common (van Kanten et al., 2005; Dias et al., 2007).

Our objectives were to (1) describe the magnitude of root trait variation within individuals of the same genotype across a range of organizational levels (site, management, and individuals); (2) evaluate which nested level of biological organization best explain root trait variation in *C. arabica*; (3) determine if root traits coordinate along a single axis of resource acquisition to resource conservation, an intraspecific RES; and (4) test whether or not an intraspecific RES is related to other well-known dimensions of ITV. Based on prior studies of root trait variation, as well as studies in intraspecific leaf trait variation in coffee, we hypothesized that root traits would vary most widely across sites (Gagliardi et al., 2015; Martin et al., 2017), which represent major climatic gradients of coffee growing conditions. Based on the emerging literature on trait ITV and within-species economic spectra (Niinemets, 2015; Martin et al., 2017), we also hypothesized that root traits would covary within coffee, along a single primary intraspecific RES; more specifically, we anticipated that root traits associated with resource acquisition trade-off with traits associated with resource conservation. Lastly, since leaves and roots respond differently to environmental gradients, we hypothesized that intraspecific root and leaf economic spectra in coffee would show little coordination among one another, and instead show patterns of orthogonality.

## Materials and Methods

### Sampling Design

Our study employed a nested design in order to quantify root and leaf trait variation in *C. arabica* plants across four different hierarchical scales: (1) individuals within blocks, (2) among blocks within management treatments (i.e., full sun and agroforestry); (3) among management treatments within a site; and (4) among sites. This design resulted in traits measured on 64 coffee plants, which were collected from *n* = 4 coffee plants per block, within *n* = 2 blocks per management treatment, within *n* = 2 management treatments per site, and across *n* = 4 sites.

We identified four sites in the coffee growing regions of Costa Rica and Nicaragua where closely related varieties of *C. arabica* (var. Caturra and var. Pacas, respectively, both derived from var. Bourbon) are grown (hereafter referred to as, collectively, *C. arabica*). While *C. arabica* var. Pacas is technically a different variety, this genotype is long-recognized only as a mutation of the same genetic strain as *C. arabica* var. Caturra (Bertrand et al., 1999). Given this mutation, there is a possibility of slight genetic variability between the two highly related varieties (but see **Table 5** for evidence of no systematic or consistent differences between varieties). These sites ranged in latitude from 9 to 11°N and elevation of 455–1500 m (covering a range of coffee growing altitudes) with a mean annual temperature (MAT) from 18.7 to 24°C and mean annual precipitation (MAP) from 1386 to 3200 mm (**Table 1**). These four sites represented four broad coffee growing regions as outlined in **Table 1**.

**Table 1:** Site and management information for farms employed in this study.

Site	Country and Coordinates	Elevation	MAP/MAT	Variety/shade tree (density)	Management treatment	Soil N	Soil C	Soil P	Soil pH	Soil moisture
Cool and wet (CW)	Costa Rica	1020	3014/19.5	Caturra/*E. poeppigiana* (6300)	Full sun	1.07 ± 0.04	10.53 ± 0.39	4.95 ± 0.59	4.23 ± 0.10	71.46 ± 2.90
	09°56′19″ N									
	83°43′46″ W									
					Agroforestry	0.96 ± 0.08	9.47 ± 0.88	9.63 ± 1.93	4.00 ± 0.10	71.85 ± 7.30
Hot and wet (HW)	Costa Rica	685	3200/23.4	Caturra/*E. poeppigiana* (5000)	Full sun	0.43 ± 0.03	4.38 ± 0.38	11.19 ± 1.12	4.38 ± 0.08	35.01 ± 1.38
	09°53′44″ N									
	83°40′7″ W									
					Agroforestry	0.37 ± 0.03	4.02 ± 0.30	7.74 ± 1.33	4.55 ± 0.13	36.45 ± 2.70
Cool and dry (CD)	Costa Rica	1500	1491/18.7	Caturra/*E. poeppigiana* (6860)	Full sun	0.26 ± 0.02	2.83 ± 0.25	4.98 ± 1.59	4.66 ± 0.08	13.39 ± 1.39
	09°40′03″ N									
	83°06′32″ W									
					Agroforestry	0.41 ± 0.01	4.66 ± 0.17	6.56 ± 1.60	4.68 ± 0.05	17.16 ± 2.74
Hot and dry (HD)	Nicaragua	455	1386/24.0	Pacas/*I. laurina* (4000)	Full sun	0.54 ± 0.05	6.10 ± 0.55	4.39 ± 1.05	4.51 ± 0.15	22.84 ± 1.54
	11°53′54″ N									
	86°08′56″ W									
					Agroforestry	0.57 ± 0.02	6.40 ± 0.20	3.35 ± 1.13	4.39 ± 0.19	23.28 ± 1.41


*We show qualitative classifications of the different study sites that coarsely approximate the range of *Coffea arabica* growing conditions, country and coordinates, elevation (m), mean annual precipitation (MAP, mm)/mean annual temperature (MAT, °C), *C. arabica* variety, shade tree (*Erythrina poeppigiana* or *Inga laurina*) and density (stems ha^-*1*^), and soil metrics at the management treatment (full sun or agroforestry) level [soil N (%), soil C (%), soil P (mg kg^-*1*^), soil pH and soil moisture (%); *n* = 16)].*

*Coffea arabica* is cultivated in two main management systems: monoculture (hereafter “full sun” management) and in agroforestry systems where coffee is intercropped with regularly pruned N_2_-fixing shade tree (hereafter “agroforestry”). For the agroforestry management system, coffee is specifically intercropped with *Erythrina poeppigiana* (Fabaceae) at three of these sites, and *Inga laurina* (Fabaceae) at one site. Within each site-by-management combination, we delineated two 25-m^2^ blocks that were spaced at minimum 20 m from one another to ensure spatial interspersion of sampling. Within each block, we selected four sample plants for collection of traits where *n* = 64 coffee plants total distributed equally among sites. In sum, all of the traits measured on coffee plants were sampled and associated with the following information: (i) roots/leaves within plants; (ii) plants within blocks; (iii) blocks within management treatments; and (iv) management treatments within sites.

At all of the sites coffee plants are stump pruned approximately every 5–7 years leading to uneven-aged canopies of coppiced resprouts, with generally 2–3 resprouts per plant (Charbonnier et al., 2013). All sampled plants were at reproductive maturity, were between 140 and 235 cm in height with a resprout basal diameter between 14.4 and 34.6 mm. In the monoculture treatments, coffee plants were at minimum 20 m away from the nearest shade tree, a distance that is excess of the zone of influence for certain physiological processes including N transfer (Meylan, 2012). In the agroforestry treatments, all sample plants were between 0.5 and 9.6 m from the nearest shade tree.

### Root Trait Collection

We excavated one complete lateral root from each individual *C. arabica* plant in our study within 1 week in order to minimize any growing season effects. The *C. arabica* root system is typically composed of a primary taproot and lateral roots in the upper soil horizon as well as four to eight axial roots (Garriz, 1978). We followed the main aerial stump to the taproot until the upper-most lateral roots could be isolated. A lateral root was then excavated in its entirety, which included all feeder and fine roots and any gravitropically positive roots. Multiple levels of standardization were used in our collection of lateral roots. Specifically, we: (1) standardized all plants by genotype (described above); (2) standardized all plants by age (based on known relationships between diameter at sprout and age); (3) standardized all intact lateral roots by depth at which they were collected (top 20 cm); and (4) standardized all intact lateral roots by originating diameter class (<4 mm). Roots were then brushed, stored intact in freezer bags, and stored at -18°C until processing. We also collected an intact soil core (5 cm diameter) from the top 20 cm of the soil profile, bagged the collected soil and roots and stored at -18°C until further processing.

In the lab, intact lateral roots were rinsed by hand using deionized water. We restricted analysis to absorptive fine roots <2 mm in diameter (Perez-Harguindeguy et al., 2013), however, given our standardization in sampling protocol described above, we included higher ordered roots (4th order but <2mm) from our intact lateral root sample. (Note – we also replicated all of our analysis on a subset of roots that fall under the “absorptive” functional classification as described by McCormack et al. (2015), which includes only 1st, 2nd, and 3rd order roots. Our results were robust with respect to this data subsetting). Samples were then scanned using a flatbed scanner at 600 dpi. Root image analysis was then conducted using WinRhizo (Regents Instruments, Montreal, QC, Canada), to generate information on total root length, total root area, total tip number, and mean root diameter. Roots from the soil cores were extracted in a water bath with tweezers and scanned for length. All roots were then oven-dried at 60°C for 48 h and weighed, and then transported to the University of Toronto Scarborough. Chemical trait analysis was conducted on the intact roots of <2 mm, which were first ground into a homogeneous powder using a ball mill (Retsch Ltd., Haan, Germany). Root C and N concentrations were then measured on approximately 0.1 g of dried sample using a CN628 elemental analyzer (LECO Instruments, Mississauga, ON, Canada).

Based on these analyses, we derived data on seven root traits in total for each plant including two traits associated with resource conservation – average root diameter (D; mm) and root carbon:N ratios (CN_root_), four traits associated with resource acquisition – SRL (root length divided by root dry mass; m g^-1^), SRA (root area divided by root dry mass; m^2^ kg^-1^), SRTD (number of root tips divided by root dry mass; tips g^-1^), root N concentrations (N_root_; %), and one trait derived from soil cores, root length density (RLD, total root length in a known soil volume; cm cm^-3^ soil). In sum, we present one trait on a standardized volume basis (RLD), three traits as a mean of <2 mm fine roots (D, N_root_, and CN_root_), and three traits on a standardized mass basis for <2 mm fine roots (SRL, SRA, and SRTD).

### Soil Conditions

Across all four sites, soil samples to a depth of 15 cm were collected and analyzed for soil properties (*n* = 64; 16 samples per site). Fresh soil samples were homogenized and divided into three for determination of soil moisture content (%), soil pH, and soil nutrients. For one of the subsamples, wet mass was measured, soils were then oven-dried at 105°C for 72 h, and soil moisture content was calculated as the difference between wet and dry mass divided by dry mass. Soil pH was measured with one of the subsamples in a 1:5 soil to water solution with a pH meter (Mettler Toledo pH meter, Mississauga, ON, Canada). The final subsample was air-dried and transported to the University of Toronto for total C and N concentrations (%), as well as available plant-available phosphorus (P, mg kg^-1^ soil). Total soil C and N concentrations were measured on approximately 1 g of dried sample using a CN628 elemental analyzer (LECO Instruments ULC, Mississauga, ON, Canada). For plant-available P determinations, samples were air-dried and sieved to 2 mm, and 4 g were extracted using 20 mL of Brays 1 and filtered through #1 Whatman filter paper. Plant-available P was then determined colorimetrically using a QuikChem8500 flow injection analyzer (Lachat Instruments, Loveland, CO, United States). Soil total N ranged from 0.34 to 1.02%, soil C ranged from 3.75 to 10.03%, soil available P ranged from 3.9 to 9.5 mg kg^-1^, soil pH ranged from 4.12 to 4.67, and gravimetric soil moisture content ranged from 15.3 to 71.6% (**Table 1**).

### Leaf Trait Data

To test the hypothesis that root and leaf trait represent independent axes of variation among coffee plants, we used a modified version of the leaf trait dataset collected by Martin et al. (2017). In short, their study was designed to quantify intraspecific variation in LES traits among the same coffee plants that were sampled here for root traits. Their dataset entailed eight traits measured on 384 coffee leaves, which corresponds to leaf traits being measured on six leaves for each coffee plant in our sample (Martin et al., 2017). To merge their dataset with ours, a plant-level average value of four leaf traits [maximum photosynthetic rates on a mass basis (*A*_mass_, μmol CO_2_ g^-1^ s^-1^); leaf mass per area (LMA, g m^-2^); leaf N concentrations (N_leaf_, %); leaf tissue density (g cm^-3^)] was calculated for analysis here.

### Statistical Analysis – Intraspecific Root Trait (Co)variation

All statistical analyses were performed using R v. 3.0.2 (R Foundation for Statistical Computing, Vienna, Austria). In total, our dataset entailed 62 observations for each root trait (two observations were removed as outliers). For each root trait, we first described the magnitude of intraspecific variation by calculating coefficients of variation (cv) across the entire dataset. We then employed a maximum likelihood approach to fit both normal and log-normal distributions to each root trait dataset, and compared models based on log-likelihood ratios. Where traits were best-described by log-normal distributions, log-transformed data was used in further analyses.

We used standardized major axis (SMA) regression models performed using the ‘lmodel2’ R package (Legendre, 2014) to examine bivariate relationships between all seven root traits (where *n* = 62 in all SMA models). For these pairwise tests, SMA were employed since we were primarily interested in the slopes of the relationships between any two given traits, all variables were assumed to be measured with error, and we did not have *a priori* hypotheses regarding the causal relationship between any pair of traits (Warton et al., 2006). Lastly, to evaluate root trait relationships in multivariate trait space, we performed a principal component analysis (PCA) using all seven root traits with the ‘vegan’ R package (Oksanen et al., 2016). Based on these analyses, we calculated PCA axis 1 and 2 scores for each root, and included these in analyses on variance decomposition (detailed below).

### Statistical Analysis – Causes of Root Trait Variation

We used a nested analysis of variance (ANOVA) coupled with variance partitioning techniques, to evaluate how categorical environmental or management (full sun or agroforestry) factors explained variation in coffee root traits. This was done by first using the ‘lme4’ R package (Bates et al., 2014) to fit a linear mixed model for each trait individually, as well as both PCA axis 1 and 2 scores. In these models, all three nested levels (i.e., block within management within site) were entered as sequential random effects, and the intercept was the only fixed effect. We then used the ‘varcomp’ function in the ‘ape’ R package (Paradis et al., 2004) to calculate the variance components associated with each nested level. These analyses were based on log-transformed data for all root traits except N_root_ and PCA axis 1 and 2 scores (**Table 2**).

**Table 2:** Intraspecific variation in roots traits of *C. arabica*.

Root trait	Log-likelihood values		Descriptive statistics		
		
	Normal	Log-normal	Mean (± s.d.)	Range	Intraspecific cv (%)
D	-10.56	-**5.86**	1.17 (0.29)	0.67–1.93	24.69
SRL	-133.56	-**121.78**	2.87 (2.10)	0.37–9.97	73.23
SRA	-423.43	-**395.88**	277.2 (255.6)	63.6–1538.9	81.39
SRTD	-449.68	-**428.67**	397.1 (344.5)	29.6–1987.2	86.76
RLD	-96.59	-**91.22**	1.53 (1.16)	0.09–5.17	75.55
N_root_	-**35.78**	-37.65	1.71 (0.43)	0.87–2.87	25.46
CN_root_	-223.47	-**216.70**	28.58 (8.97)	14.14–53.69	31.38


*The maximum likelihood-based model that best-describes the distribution of each trait is highlighted in bold (where *n* = 62 in all cases). Root traits are: root diameter (D; mm), specific root length (SRL; m g^-*1*^), specific root area (SRA; m^*2*^ kg^*1*^), specific root tip density (SRTD; tips g^*1*^), root length density (RLD; cm cm^-*3*^), root nitrogen concentrations (N_*root*_; %), and root carbon:N ratios (CN_*root*_).*

For each trait, we then performed an additional mixed model analysis that included soil pH, soil N, soil C, soil P, and soil moisture as fixed effects, and our three nested categorical factors (site, management, and block) as random effects. For these models we then calculated the proportion of intraspecific root trait variation explained by the fixed effects alone (i.e., the “fixed effects *r*^2^”) and the proportion of ITV explained by both the fixed and random factors combined (i.e., the “fixed effects + random effects *r*^2^”) (Nakagawa and Schielzeth, 2013). These values were calculated using the ‘sem.model.fits’ function in the ‘piecewiseSEM’ R package (Lefcheck, 2016).

### Statistical Analysis – Intraspecific Root and Leaf Trait Coordination

To test if root and leaf traits represent independent axes of variation in *C. arabica*, we used a MFA: a multivariate ordination method that tests if different groups of variables form independent structures within a dataset. This analysis follows previous studies testing the independence of trait spectra across species (c.f. Baraloto et al., 2010), but in our case, the MFA tests if root traits covary and leaf traits covary independently from one another within *C. arabica*. The test statistic derived from our MFA is the RV coefficient that describes the relationship among root and leaf trait spectra; values closer to zero indicate little correlation among leaf and root traits, and coefficients approaching an absolute value of 1 indicate stronger relationships among leaf and root trait dimensions. We used a permutation test to generate a one-tailed significance value for our RV coefficient. Specifically, root and leaf traits were determined to vary independently from one another, if our observed RV coefficient fell within the lower 95% percentile of the distribution of RV coefficients, derived from *n* = 10000 permuted datasets. In order to test if our observed patterns of coordination/decoupling among leaf and root trait spectra were heavily influence by a single root trait that did not strongly align with an RES (see results below), this MFA analysis and permutation test was also performed on a dataset with RLD removed. All MFA tests were performed using the “FactoMineR” R package (Le et al., 2008).

## Results

### Root Trait (Co)variation

All *C. arabica* root traits exhibited considerable intraspecific variation, with cvs ranging from approximately 25–87% (**Table 2**). The two root chemical traits, N_root_ and CN_root_, exhibited low variation at 25.46 and 31.38%, respectively, while root D also exhibited low intraspecific variation (cv = 24.69 %). All other traits varied by over an order of magnitude within *C. arabica*, with cvs ≥ 73%. In particular, SRL ranged from 0.37 to 9.97 m g^-1^, SRA from 63.6 to 1538.9 m^2^ kg^-1^, and RLD from 0.09 to 5.17 cm cm^-3^ (**Table 2**). Specific root tip density exhibited the highest intraspecific variation (cv = 86.76%), with SRTD values ranging over almost two orders of magnitude from 29.6 to 1987.2 tips g^-1^ (**Table 2**).

### Bivariate Root Trait Correlations

Pairwise relationships between all *C. arabica* root traits were strong and highly significant (**Table 3**). Specifically, three root morphological traits that are associated with resource acquisition (i.e., SRL, SRA, and SRTD) were strongly and significantly positively related with one another (*r*^2^ = 0.479–0.804, *p* < 0.001 for all three tests), while root D was negatively related with SRL (*r*^2^ = 0.645; *p* < 0.001), SRA (*r*^2^ = 0.497; *p* < 0.001), and SRTD (*r*^2^ = 0.501; *p* < 0.001). The root chemical trait associated with resource acquisition, N_root_, was significantly positively related with SRL, SRA, SRTD, and RLD, (*r*^2^ = 0.172–0.431; *p* < 0.001), and significantly negatively related with root D (*r*^2^ = 0.226; *p* < 0.001).

**Table 3:** Bivariate relationships among seven root traits in *C. arabica*.

	log-D	log-SRL	log-SRA	log-SRTD	log-RLD	N_root_	log-CN_root_
log-D	—	-0.212(-0.341, -0.251)	-0.361(-0.434, -0.301)	-0.273(-0.328, -0.228)	-0.229(-0.288, -0.183)	-1.846(-2.312, -1.473)	0.8008(0.644, 0.995)
log-SRL	0.645 (<0.001)	—	0.809(0.700, 0.934)	0.935(0.834, 1.048)	0.784(0.626, 0.982)	1.845(1.528, 2.250)	-2.740(-3.323, -2.260)
log-SRA	0.497 (<0.001)	0.687 (<0.001)	—	0.756(0.628, 0.910)	0.634(0.503, 0.800)	0.667(0.538, 0.824)	-2.216(-2.731, -1.798)
log-SRTD	0.501 (<0.001)	0.804 (<0.001)	0.479 (<0.001)	—	0.839(0.658, 1.068)	1.983(1.617, 2.431)	-2.930(-3.582, -2.397)
log-RLD	0.209 (<0.001)	0.227 (<0.001)	0.180 (<0.001)	0.105 (0.005)	—	2.364(1.873, 2.984)	-3.494(-4.394, -2.778)
N_root_	0.226 (<0.001)	0.431 (<0.001)	0.318 (<0.001)	0.369 (<0.001)	0.172 (<0.001)	—	-1.478(-1.630, -1.340)
log-CN_root_	0.279 (<0.001)	0.436 (<0.001)	0.335 (<0.001)	0.387 (<0.001)	0.198 (<0.001)	0.856 (<0.001)	—


*All relationships are based on standardized major axis regression analysis, where *n* = 62 for all tests. The upper right portions of the table represent the slopes of bivariate trait relationships with associated 95% confidence limits (in brackets). The bottom left portion represents *r*^*2*^ values and associated *p*-values (in brackets).*

### Multivariate Root Trait (Co)variation

**FIGURE 1:**
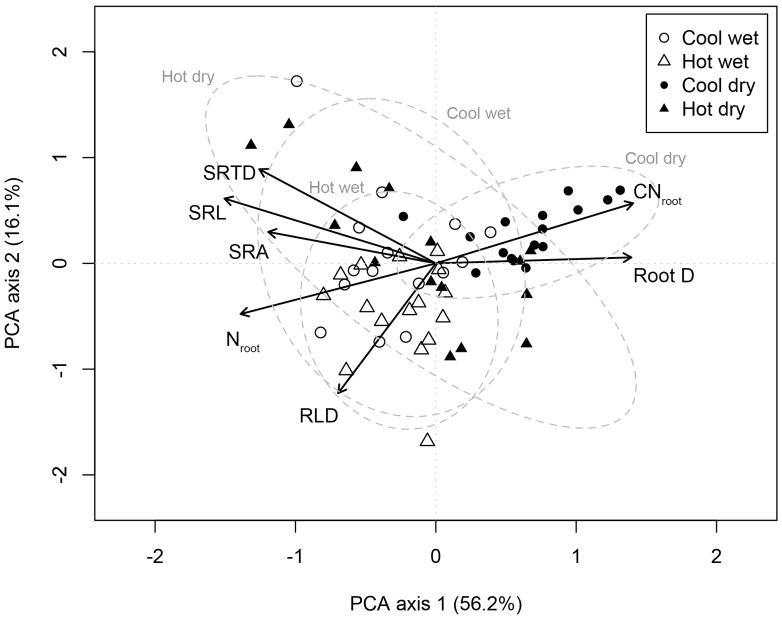
Principal components analysis of intraspecific variation in root functional traits in *Coffea arabica*. The PCA is based on root traits measured on 62 individual-plants, sampled across four different sites (open circles = CW, open triangles = HW, closed circles = CD, and closed triangles = HD). Dashed lines correspond to 95% confidence ellipses for *C. arabica* roots sampled at each site. PCA axis 1 scores were significantly different (*p* < 0.001) for CD as compared to CW, HW, and HD.

The first two PCA axes explained 72.3% of the variation in the seven root traits among *C. arabica* plants (**Figure 1**). PCA axis 1 explained the majority of this variation (56.2%) and was significantly positively associated with D (*p* < 0.001) and CN_root_ (*p* < 0.001) while negatively associated to root traits associated with resource acquisition, SRL (*p* < 0.001), SRA (*p* < 0.001), SRTD (*p* < 0.001), RLD (*p* = 0.001), and N_root_ (*p* < 0.001) (**Figure 1**). PCA axis 1 highlights the trade-offs between traits associated with resource acquisition and traits associated with resource conservation; PCA axis 1 scores were also significantly (*p* < 0.001) related to site category. PCA axis 2 explained a further 16.1% of the variation in the seven root traits among *C. arabica* plants (**Figure 1**).

### Variance Partitioning

For all root traits, the site in which a *C. arabica* plant was growing consistently explained the majority of intraspecific variation among plants (**Table 4**). Specifically, among morphological traits, site identity explained 29.1% of the variance in root D, 68.3% of the variance in RLD, and 36.1% of the variance in SRL (**Table 4**). Intraspecific variation in root chemical traits was also best explained by site (N_root_ = 27.4%; CN_root_ = 42.1%), as compared to management treatment or block. Management treatment did explain the largest proportion of variance in SRTD (35.4%) and SRA (29.1%), but site identity explained an additional 21.4 and 16.9% of the variation, respectively. Management treatment also explained between ∼15 and 28% of the variation in SRL, N_root_, and CN_root_ (**Table 4**). Site and management also explained root trait variation in multivariate trait space. Specifically, 33.7 and 31.7% of the variation in PCA score 1 was explained by site and management, respectively, while 4.1 and 39.4% of the variation in PCA score 2 was explained by site and management, respectively (**Table 4**). Within the exception of N_root_, block identity explained <11% of the intraspecific variation in any coffee root trait (**Table 4**).

**Table 4:** Sources of intraspecific variation in root traits of *C. arabica*.

Root trait	Variance decomposition				Mixed effects model	
		
	Site	Management	Block	Error	Fixed effects *r*^2^	Fixed effects + random effects *r*^2^
log-D	**29.1**	18.7	8.4	43.9	0.0463	0.6158
log-SRL	**36.1**	27.5	3.2	33.2	0.0721	0.6864
log-SRA	16.9	**29.7**	10.9	42.4	0.0660	0.6337
log-SRTD	21.4	**35.4**	2.0	41.2	0.1045	0.6259
log-RLD	**68.3**	6.7	3.2	21.8	0.0181	0.7944
N_root_	**27.4**	15.6	16.2	40.8	0.0434	0.7351
log-CN_root_	**42.1**	24.1	2.7	31.1	0.0245	0.7479
PCA1	**33.7**	31.7	5.4	2.9	0.0148	0.7432
PCA2	4.1	**39.4**	5.4	51.1	0.0846	0.4697


*Variance decomposition was based on a nested analysis of variance, which for each trait was based on *n* = 62 individual-plants. The nested level explaining the highest percentage of variation in a given trait is highlighted in bold. Also presented are the explained variance associated with continuous soil variables (soil N, soil C, soil P, soil pH, and soil moisture) measured within these nested levels (“Fixed effects *r*^*2*^”), and the explained variance associated with both the fixed effects and random effects.*

Continuous soil parameters (soil N, soil C, soil P, soil pH, and soil moisture) measured within the nested levels also explained very little of the variance in any root traits (**Table 4**). Specifically, these continuous variables explained only between 1.5 and 10.5% of the variation in any root trait, indicating that continuous soil variables did not systematically explain intraspecific root trait variation. In comparison, the addition of the nested categorical levels to mixed models increased the explanatory power to 47–79% (**Table 4**).

Given that the nested level “site” strongly explained variance in root traits, we conducted an ANOVA of root traits among sites (**Table 5**). Root D, SRL, and RLD were significantly different at the CD site as compared to the other three sites (**Table 5**); larger root D was paired with lower SRL and SRA at the CD site. Similarly, N_root_ and CN_root_ were significantly different at this site, expressing lower concentrations of N and higher C:N ratios (**Table 5**).

**Table 5:** Mean (± s.d.) of root traits among the four sites.

Trait	Cool and wet (CW)	Hot and wet (HW)	Cool and dry (CD)	Hot and dry (HD)
D	1.12 (0.13)^a^	1.04 (0.14)^a^	1.47 (0.30)^b^	1.05 (0.31)^a^
SRL	4.01 (2.06)^a^	2.98 (1.29)^a^	1.05 (0.80)^b^	3.56 (2.62)^a^
SRA	329.1 (156.2)^ab^	289.0 (109.7)^ab^	131.8 (63.5)^a^	367.5 (379.7)^b^
SRTD	607.4 (426.0)^ab^	378.5 (195.3)^ab^	160.6 (121.3)^a^	458.9 (404.0)^b^
RLD	1.74 (0.82)^a^	2.22 (1.16)^a^	0.30 (0.22)^b^	1.90 (1.10)^a^
N_root_	1.96 (0.44)^a^	1.94 (0.33)^a^	1.34 (0.34)^b^	1.59 (0.31)^b^
CN_root_	24.61 (4.52)^a^	21.36 (3.17)^a^	37.23 (9.85)^b^	31.02 (6.83)^b^
PCA 1 score	-0.31 (0.39)^a^	0.26 (0.29)^a^	0.66 (0.38)^b^	-0.11 (0.63)^a^
PCA 2 score	0.05 (0.61)^a^	-0.45 (0.45)^b^	0.30 (0.26)^a^	0.11 (0.68)^a^


*Values followed by the same letter within a row are significantly different based on Tukey’s HSD test (*n* = 16).*

### Root and Leaf Trait Coordination

**FIGURE 2:**
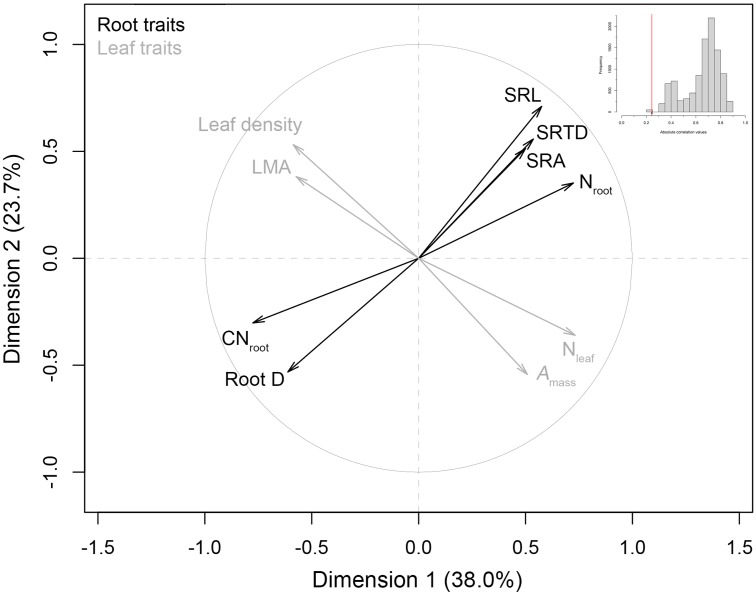
Multiple factor analysis of root traits and leaf economics traits in *C. arabica*. Leaf traits were based on tree-level values derived from Martin et al. (2017), and *a priori* multiple factor analysis (MFA) groupings of root traits [with the removal of root length density (RLD)] and leaf traits, are denoted by gray and black coloring, respectively. A histogram of the randomized absolute correlation values (RV values) between these trait groups (where *n* = 10, 000 randomizations) is also presented as an inset. The red line indicates the observed correlation (RV) between root and leaf groups, relative to the distribution expected if traits were randomly assigned to groups.

Multiple factor analysis indicated a significant lack of intraspecific coordination among *C. arabica* root traits and leaf traits (**Figure 2**). These two trait spectra were strongly orthogonal to one another, with an observed RV correlation coefficient of 0.245 that ranked as the lowest observed RV value in our randomized distribution of RV coefficients (randomization test *p* < 0.001, **Figure 2** inset). These MFA results were robust with respect to the inclusion of RLD: the one root trait that is measured on a per soil volume basis and aligns most closely with axis 2 of our RES PCA (see **Figure 1**). Specifically, when RLD is included in the analysis, the MFA RV = 0.244 (*p* = 0.02) (data not shown). Qualitatively, *A*_mass_, N_leaf_, LMA, and leaf tissue density covaried along an LES, while root traits covaried along an independent trait spectrum (**Figure 2**).

## Discussion

### The Existence an Intraspecific Root Economics Spectrum

Our findings contribute to the growing literature evaluating how trait spectra commonly used to describe interspecific differences in functional biology, may also describe the ecological variability among plants within a species (Hajek et al., 2013; Gagliardi et al., 2015; Niinemets, 2015). Although there remains limited evidence that species in fact differentiate across a universal RES (Weemstra et al., 2016), we did find strong support for a single, well-defined RES in *C. arabica*. Specifically, this intraspecific RES describes bi- and multivariate trade-offs among resource acquiring traits on one hand – namely high values of SRL, SRA, SRTD, RLD, and N_root_ – and resource conservation traits on the other – namely high values of CN_root_ and root D (**Figure 1**).

The relationship among root D and specific root length and area measures must be tempered with an inherent autocorrelation to SRL and SRA (Weemstra et al., 2016), and controversy in regards to the meaningfulness of these relationships (van Kanten et al., 2005). However, the trade-off between root D with SRTD and N_root_ as well as SRL, SRA, and RLD with CN_root_ provides substantive evidence of a singular axis. We indicate strong covariation across root acquisition and conservative traits, with coffee plants assembling along a hypothesized resource acquisitive-conservative spectrum. Our results also suggest that when evaluating the traits comprising a RES, including all traits that are strongly tied to resource acquisition potential (c.f. Cahill et al., 2010), may increase the ability to detect a RES within species.

### The Role of Abiotic Gradients and Biotic Interactions in Forming an RES

In our study, site identity explained the largest portion of variance for most of our root traits for five of the seven traits, while management systems (full sun versus agroforestry) explained the largest portion of variance for two of the seven traits (SRA and SRTD) (**Table 4**). Multivariate trait syndromes differed most consistently according to site-level differences. We show that PCA axis 1 scores were significantly different for the cold and dry site as compared to the other three sites (*p* < 0.001; **Figure 1** and **Table 5**). Generally, root traits for plants growing at the CD site were significantly different than those at the other sites (**Table 5**), tending toward the resource conservative end of the intraspecific RES (notably, higher D, lower N_root_, higher CN_root_); differences that appear largely attributable to the significantly lower soil moisture content at the CD site (**Table 1**), but could be due to a range of abiotic factors not captured in this study. This conforms to previous suggestions that plant traits will hinge on environmental gradients, highlighting the need to replace species mean traits with distributions that can be used to describe the breadth of ITV (Albert et al., 2010).

Significant relationships between root traits and soil fertility metrics have been demonstrated at the plant community level (Fort et al., 2016) and across land-use types (Prieto et al., 2015), though results are not consistent; for example, SRL has been observed to both increase (Fort et al., 2016) and decrease (Prieto et al., 2015) with increasing soil fertility. Similar inconsistencies have been shown in other studies on interspecific variation in root trait: Holdaway et al. (2011) found that species grown in P limited environments exhibited high SRL, low root D, and high root branching, while Ostonen et al. (2007) found that SRL decreases with increasing fertilization. Although Tobner et al. (2013) illustrate that intraspecific variation in certain root traits, namely root D, may indicate environmental change, our continuous soil variables (soil N, soil C, soil P, soil pH, and soil moisture) embedded within our nested levels explained little ITV (1.5–10.5%) in *C. arabica* roots (**Table 4**), and were not sufficient to capture the full range of explanatory soil variables. Highly dynamic soil metrics, such as N mineralization rates or hydraulic conductivity may be more well suited to predict root ITV at the site- through to the individual-plant level, however, this is beyond the scope of this study.

Unmeasured components of the root economy in ecosystems may also be more adept in explaining ITV in roots. For example, Valverde-Barrantes et al. (2016) suggest an ‘alternative adaptation’ to resource acquisition leading to higher colonization space for arbuscular mycorrhizal fungi (AMF), which results in root traits that defy strict trade-offs along a RES. Furthermore, associations to AMF versus ectomycorrhizal fungi among woody species may differentially impact root morphology, root elongation, and tip density (Chen et al., 2016). In our study systems, the most significant feature differentiating the two management systems is the presence or absence of N_2_-fixing Fabaceae trees, which consequently results in alternate pathways for N deposition (Munroe and Isaac, 2014). Whereas the presence of an N_2_-fixing tree has a strong influence on *C. arabica* leaf chemical traits (Martin et al., 2017), but not roots (**Table 4**), much remains unknown about the impact of N_2_-fixing trees on the N economy of neighboring plants as well as fungal communities within these systems, two potential drivers of root trait form and function.

### Intraspecific RES and LES Orthogonality

Our findings suggest that orthogonality of trait spectra that has been observed across species (Baraloto et al., 2010; Li et al., 2015), also describe the relationship between an intraspecific RES and LES in *C. arabica* (**Figure 2**). These two axes of variation are independent of one another, suggesting that within our study species, root traits vary in response to environmental conditions that do not necessarily result in commensurate changes in leaf form and function. These results support recent work on the lack of coordination between a RES and other trait spectra across species (Weemstra et al., 2016), but it is important to note that interspecific studies on root trait coordination/orthogonality with other known dimensions of plant functional specialization, have produced conflicting results (e.g., Liu et al., 2010; de la Riva et al., 2016).

One explanation for the decoupling of RES and LES traits within a species observed here, is that root traits are less constrained than leaves in terms of ‘phenotypic morphospace’ (Donovan et al., 2014). Individual phenotypic leaf plasticity is constrained while root trait expression exhibits larger variability. These differences are despite strong phylogenetic constraints on root traits that should limit their variability (Cornwell et al., 2014; Kramer-Walter et al., 2016). *C. arabica* plants exhibited relatively low variation in leaf morphological (LMA cv = 17.3%) and chemical traits (leaf C and N cv ≤ 10.1%, Martin et al., 2017), as compared to analogous root traits (SRL cv = 73.23%; SRA cv = 81.39%; N_root_ cv = 25.46%; **Table 2**). This may be due to greater differentiation as a result of the multifunctional nature of roots for resource acquisition in a highly heterogeneous space as well as structural support. Furthermore, this lack of constrained ITV may be particularly strong in agroecosystems, given management practices that increase resource (water and nutrients) stability and thus allow for greater niche differentiation.

An alternative explanation engages the role of artificial selection (Milla et al., 2015; Martin et al., 2017). Given luxury resource availability in agroecosystems, artificial selection has been hypothesized to shift crop trait values toward the resource acquisition end of any trait spectra (Milla et al., 2014; Gagliardi et al., 2015). Additional studies testing hypotheses on how root trait syndromes have evolved across species are needed (Valverde-Barrantes and Blackwood, 2016). It is possible that selection has resulted in trait trade-offs in *C. arabica* that are less strong as compared to those observed in wild plants, but comparative analyses (c.f. Figure 2 in Martin et al., 2017) would be needed to test this expectation.

## Conclusion

Observations that an intraspecific RES in *C. arabica* is orthogonal to an intraspecific LES, contributes to our understanding of the causes of whole plant ITV and will advance our predictive ability of *C. arabica* productive vigor and response to environmental change. Recent analyses point to a lack of root functional trait data as a major limitation when predicting the effects of climate change on yield or other agroecosystem functions (Rosenzweig et al., 2014). On one hand, our results indicate that root trait data cannot be reliably approximated based on correlations with other traits that might be more easily measured. However, our results here suggest root trait data from different sites or management-systems, may indeed capture the large majority of ITV in root traits, and could be incorporated into local-level models of agroecosystem function. For example in *C. arabica*, certain models (e.g., van Oijen et al., 2010) incorporate CN_root_ data as a predictor of yield and C storage; our results show that nearly 70% of the total variation in this particular trait can be accounted for by sampling *C. arabica* roots across sites and management-systems. As quantifying ITV in crops remains a key data requirement for many of the world’s most widely employed crop models (Bouman and van Laar, 2006), new site-specific observations of root traits will be central in refining such models.

As *C. arabica* is one of the most economically important tree-crops globally, providing evidence-based criteria to manage such systems across climatic and management conditions is undoubtedly needed for the success of coffee producers, particularly smallholder farmers. Extending our results to other important crop or wild plant species could have important implications for predicting ecosystem structure and function.

## Author Contributions

MI designed and coordinated the study, conducted data collection and analysis, and drafted the manuscript. AM assisted in study design, participated in data collection, data analysis, and manuscript writing. EdM, BR, OR, and KV contributed to site establishment, study design, and manuscript writing. All authors gave final approval for publication.

## Conflict of Interest Statement

The authors declare that the research was conducted in the absence of any commercial or financial relationships that could be construed as a potential conflict of interest.
